# *Schistosoma haematobium*–*Schistosoma mansoni* Hybrid Parasite in Migrant Boy, France, 2017

**DOI:** 10.3201/eid2502.172028

**Published:** 2019-02

**Authors:** Yohann Le Govic, Julien Kincaid-Smith, Jean-François Allienne, Olivier Rey, Ludovic de Gentile, Jérôme Boissier

**Affiliations:** Université Bretagne Loire, Angers, France (Y. Le Govic);; Centre Hospitalier Universitaire d’Angers, Angers (Y. Le Govic, L. de Gentile);; Université de Montpellier, Perpignan, France (J. Kincaid-Smith, J.-F. Allienne, O. Rey, J. Boissier)

**Keywords:** hematuria, urinary schistosomiasis, *Schistosoma haematobium*, *Schistosoma mansoni*, hybrids, hybrid parasite, migrants, parasites, France, Ivory Coast, ectopic egg elimination, *cox*1, phylogenetic analysis, internal transcribed spacer, ITS, Côte d’Ivoire

## Abstract

Schistosomiasis is frequently detected in persons entering Europe. In 2017, we detected a *Schistosoma mansoni*–*Schistosoma haematobium* hybrid parasite infection in a migrant boy from Côte d’Ivoire entering France. Because such parasites might be established in Europe, as illustrated by an outbreak on Corsica Island, vectors of these parasites should be investigated.

In 2017, a 14-year-old boy from Côte d’Ivoire who had crossed the Sahara Desert through Niger and reached Europe by sea from Libya was referred to the Consultation Board at the Parasitology Unit of Angers University Hospital (Angers, France) for painless gross hematuria. Aside from hematuria, his physical examination was unremarkable. Blood tests revealed microcytic anemia (hemoglobin 103 g/L, mean corpuscular volume 57.1 fL, mean corpuscular hemoglobin concentration 285 g/L) and a low serum ferritin level (8 µg/L). Leukocyte and eosinophil counts and biochemical markers of liver and kidney function were within reference limits. Serologic test results were negative for antibodies to HIV, hepatitis B and C virus, and *Treponema pallidum*. Serodiagnostic test results were negative for cystic and alveolar echinococcoses and strongyloidiasis, and schistosomiasis screening test results were inconclusive: positive by *Schistosoma mansoni*–specific ELISA (optical density 1.02, threshold 0.53; Bordier Affinity Products, http://www.bordier.ch/) and negative by *S. mansoni* indirect hemagglutination test (titer 1:40, threshold 1:160; Bilharziosis Fumouze; https://www.biosynex.com/).

To clarify diagnosis, we performed the SCHISTO II Western Blot IgG (LDBio Diagnostics, http://www.ldbiodiagnostics.com/), which showed 5 unequivocal bands, including a large band at 22–24 kDa and 30–34 kDa, results indicative of schistosomiasis. We corroborated our results by microscopic examination of patient feces and urine. We detected lateral-spined eggs (typical of *S. mansoni* parasites) in a fecal specimen processed by using the Kato-Katz method and, surprisingly, lateral-spined eggs and terminal-spined eggs (typical of *Schistosoma haematobium* parasites) in a 24-hour urine specimen filtered through a 12-micron membrane (Figure, panel A). The patient received a single 40 mg/kg dose of praziquantel. The patient experienced no more episodes of hematuria for the following 6 months; however, we could not assess his parasitological responses.

**Figure:**
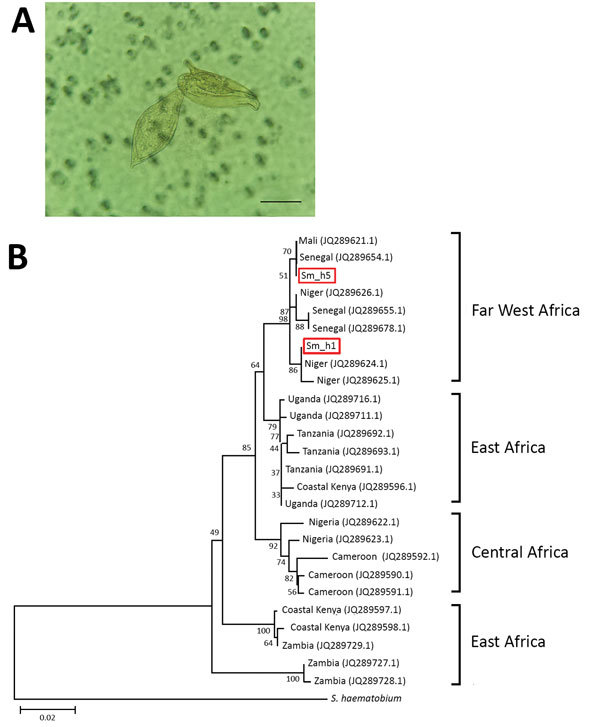
Characterization of *Schistosoma* parasites detected in 14-year-old migrant boy from Côte d’Ivoire in France, 2017. A) Co-detection of terminal-spined schistosome eggs (typical of *Schistosoma haematobium* parasites) and lateral-spined schistosome eggs (typical of *Schistosoma mansoni* parasites) in urine sample from migrant boy. Sample was microscopically examined after filtration. Original magnification ×400. Scale bar represents 50 µm. B) Phylogenetic analysis of *S. mansoni* *cox*1 gene haplotypes present in migrant boy (boxes). Tree was constructed by using the neighbor-joining method, the Hasegawa–Kishino–Yano plus gamma distribution model, and 1,000 bootstrap replicates. GenBank accession numbers of haplotypes sampled are provided. Scale bar indicates nucleotide substitutions per residue.

We genotyped both terminal- and lateral-spined eggs individually using a previously described protocol (*1*). All terminal-spined eggs were characterized by mitochondrial *cox*1 genes (GenBank accession nos. MG562514–5) and the nuclear internal transcribed spacer (ITS) (GenBank accession no. MG554667) specific to *S. haematobium* schistosomes. All chromatograms of ITS genes from lateral-spined eggs showed a double profile: 1 identical to *S. mansoni* schistosomes (GenBank accession no. MG554659) and 1 identical to *S. haematobium* (GenBank accession no. MG554667). Moreover, the *cox*1 haplotypes of these eggs were either specific to *S. haematobium* (GenBank accession no. MG562514) or *S. mansoni* (GenBank accession nos. MG562512–3) parasites. The phylogenetic tree of *S. mansoni cox*1 sequences indicates that the parasite responsible for this infection originated from Far West Africa; the hybrid parasite’s haplotypes clustered with those of schistosomes from Niger, Senegal, and Mali (Figure, panel B).

Schistosomiasis represents a serious disease burden worldwide and is ranked the 12th most common travel-associated infection in Europe (*2*). The migration crisis has led to a large flow of persons (notably children) from West Africa. Unaccompanied foreign minors are protected under the 1989 United Nations Convention on the Rights of the Child (https://www.ohchr.org/en/professionalinterest/pages/crc.aspx). Moreover, because of the high prevalence of schistosomiasis in their countries of origin, these children receive care for this disease upon their arrival in Maine-et-Loire Department (Loire Valley, France). This preventive strategy enabled us to diagnose schistosomiasis in ≈25% of travelers and migrants (Y. Le Govic, unpub. data), a result in accordance with a similar screening program in Italy (*3*). This strategy also enabled us to detect the case of mixed *Schistosoma* parasite infection with ectopic egg elimination described in this report.

Ectopic egg elimination (i.e., *S. haematobium* schistosome eggs in feces and *S. mansoni* eggs in urine) frequently occurs in endemic areas; in a study in northern Senegal, 53% of patients infected with *Schistosoma* parasites had simultaneous infections with *S. mansoni* and *S. haematobium* parasites, of which 15% displayed ectopic egg elimination (*4*). This phenomenon can occur because of parasite hybridization. Hybrids resulting from *S. mansoni* and *S. haematobium* schistosome cross-breeding have been documented in northern Senegal (*5*). The fact that the patient we describe never traveled through Senegal strongly suggests that such hybrids are more widespread than previously observed.

Hybrid schistosomes are particularly worrisome. The outbreak in Corsica Island involved infections with *S. haematobium*–*Schistosoma bovis* hybrid parasites (*1*,*6*), which might be more difficult to diagnose (*7*). Experimental studies have revealed that interspecific hybridization might enhance infectivity, virulence, and longevity and accelerate cercarial maturation. Also, hybrids can have wider host spectrums, potentially expanding their epidemiologic consequences (*8*).

The risk for emergence of *S. mansoni* schistosome infection might exist in Europe; the *Biomphalaria* snail vector (namely *B. tenagophila*) has been detected for several years in Romania (*9*). Given the nonnegligible prevalence of mansonic schistosomiasis in travelers and migrants entering Europe (*3*,*10*) and global warming, the probability of encounters between *S. mansoni* miracidia and their snail hosts might have increased. Moreover, whether *S. mansoni*–*S. haematobium* hybrid parasites are capable of infecting the *Bulinus* snail vector of the *S. haematobium* schistosome*,* which is widely distributed throughout Europe (France, Spain, Italy, Greece, Portugal) (*11*), remains unknown and deserves further investigation.
